# Supplementary material for: The Nexus between Fire and Soil Bacterial Diversity in the African Miombo Woodlands of Niassa Special Reserve, Mozambique
Source: Microorganisms. 2021 Jul 22;9(8):1562. doi: 10.3390/microorganisms9081562 (PMC8400031; doi:10.3390/microorganisms9081562)
Supplement: Supplementary file 1 [file microorganisms-09-01562-s001.zip › microorganisms-1291111-supplementary.pdf]

Figure S1

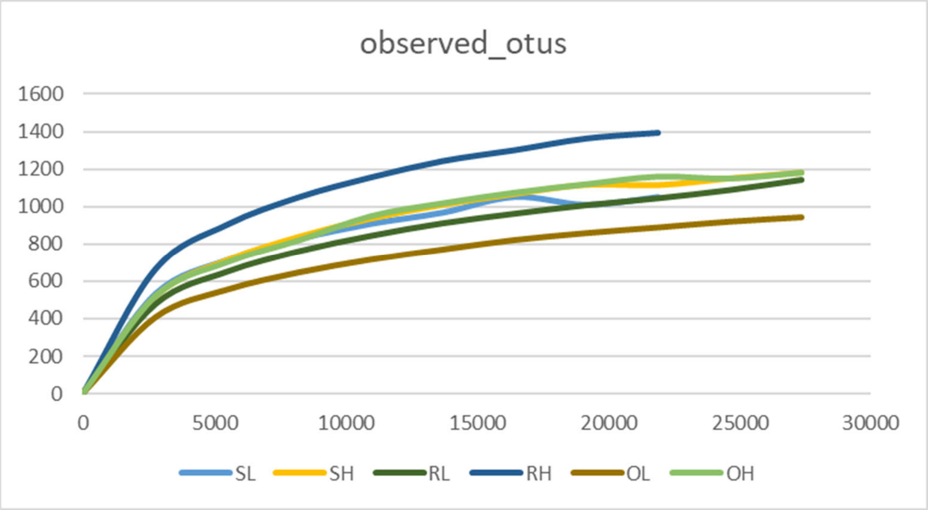

Figure S2

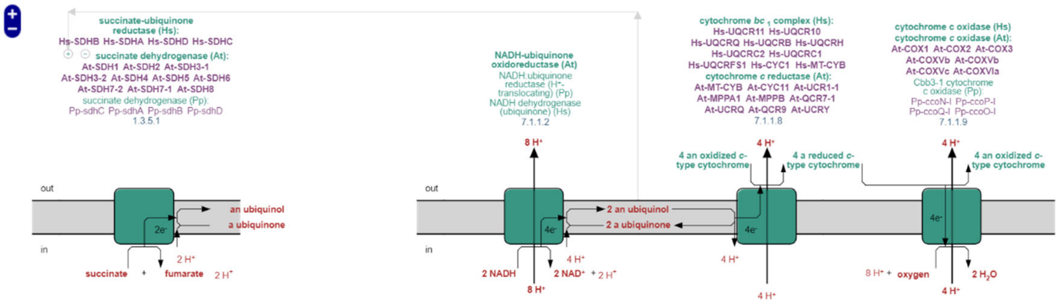

Table 1. Statistics of raw data of soil samples of Miombo woodlands. S: Brownish-gray sandy soils; R: Red soils of medium texture; O: Red soils with medium texture oxic. Soil type code followed by H indicates high fire frequency while L indicates low fire frequency.

Soil type	Fire frequency	Code	Total Bases	Read Count	N (%)	GC (%)	Q20 (%)	Q30 (%)
**Brownish-gray sandy soils**	Low	SL	434027445	959134	0	58	97	90
	High	SH	409324077	908975	0	58	98	91
**Red soils of medium texture**	Low	RL	404076937	889657	0	58	97	91
	High	RH	417281764	923287	0	58	97	91
**Red soils with medium texture oxic**	Low	OL	422193616	931887	0	57	98	91
	High	OH	370550641	816239	0	58	97	90
	Total		**2,457,454,480**	**5,429,179**		57.83		

Table S2. ANOVA results showing bacterial differences at the top Phyla between the type of soil (ST), the fire frequency (FI) and its interaction. Significant results are indicated in red.

Acidobacteria	df	MS	F	P
ST	2	35518794	7.6757	0.0038
FI	1	994301	0.4297	0.0021
FI*ST	2	1137523	0.2458	0.0541
**Actinobacteria**				
ST	2	1248980	0.7366	0.4926
FI	1	1356880	9.4450	0.0065
FI*ST	2	2323670	2.2848	0.1305
**Proteobacteria**				
ST	2	9197876	0.6693	0.5243
FI	1	2801520	0.0407	0.8422
FI*ST	2	19867546	1.4458	0.2616

Table S3. ANOVA results showing bacterial differences at the top Genus between the type of soil type (ST), the fire frequency (FI) and its interaction. Significant results are indicated in red.

Effect	df	MS	F	P
**Chthoniobacter**				
**ST**	2	20908415	18.1981	0.000048
**FI**	1	11972350	10.4204	0.004665
**FI*ST**	2	7043133	6.1301	0.009324
**Gaiella**				
**ST**	2	4414294	3.6423	0.046962
**FI**	1	3500648	4.8884	0.006211
**FI*ST**	2	1306593	5.0781	0.035100
**Solirubrobacter**				
**ST**	2	2084752	4.4237	0.027374
**FI**	1	2208873	4.6871	0.044073
**FI*ST**	2	2842036	6.0306	0.009895
**Saccharopolyspora**				
**ST**	2	345600	5.1102	0.04500
**FI**	1	123669	2.9901	0.05671
**FI*ST**	2	234891	2.5619	0.05011
**Bacillus**				
**ST**	2	348810	5.6667	0.04021
**FI**	1	133488	4.6871	0.05022
**FI*ST**	2	230090	3.2252	0.00610

Table S4. Top 20 pathways detected by PICRUSt in miombo samples.

Pathway	Description	SH	SL	RL	RH	OL	OH
PWY-3781	aerobic respiration I (cytochrome c)	57957.63	82600.85	90537.08	64231.53	99601.01	73689.28
PWY-7111	pyruvate fermentation to isobutanol (engineered)	34242.77	48153.65	51866.44	38762.56	56226.01	39516.54
PWY-5101	L-isoleucine biosynthesis II	32572.09	43776.91	49126.74	37244.49	53291.43	39323.15
ILEUSYN-PWY	L-isoleucine biosynthesis I (from threonine)	31887.65	43623.63	48314.61	36577.27	51098.10	37979.65
VALSYN-PWY	L-valine biosynthesis	31887.65	43623.63	48314.61	36577.27	51098.10	37979.65
BRANCHED-CHAIN-AA-SYN-PWY	superpathway of branched amino acid biosynthesis	27460.04	36695.92	42277.82	31742.49	46132.10	34691.72
PWY-7094	fatty acid salvage	26381.72	38646.21	47058.33	32104.45	39223.41	34424.06
NONOXIPENT-PWY	pentose phosphate pathway (non-oxidative branch)	25145.90	37994.14	40369.04	27045.89	40940.09	30867.38
PWY-5103	L-isoleucine biosynthesis III	24262.10	31682.54	36806.33	27598.97	41139.96	30443.07
PWY-6969	TCA cycle V (2-oxoglutarate:ferredoxin oxidoreductase)	23998.91	32097.75	35752.97	26459.81	40560.07	29713.41
FAO-PWY	fatty acid β-oxidation I	23696.00	34162.49	42286.14	29068.43	35961.48	31922.50
TCA	TCA cycle I (prokaryotic)	23588.44	30655.76	34264.75	25731.96	40235.69	28952.61
PHOSLIPSYN-PWY	superpathway of phospholipid biosynthesis I (bacteria)	22891.44	32093.25	33956.38	25847.17	40609.38	30144.36
PWY-5667	CDP-diacylglycerol biosynthesis I	22573.70	31594.65	32149.99	24786.99	38860.26	28767.02
PWY0-1319	CDP-diacylglycerol biosynthesis II	22573.71	31594.64	32149.98	24786.97	38860.26	28767.01
PWY-5973	cis-vaccenate biosynthesis	22280.09	26902.89	29110.97	25228.32	38502.77	27010.73
PWY-5659	GDP-mannose biosynthesis	22063.24	29674.78	35581.39	26816.63	38277.11	30623.15
PWY4FS-7	phosphatidylglycerol biosynthesis I (plastidic)	21786.33	30217.11	32346.94	24557.02	38485.80	28521.81
PWY4FS-8	phosphatidylglycerol biosynthesis II (non-plastidic)	21786.33	30217.11	32346.94	24557.02	38485.80	28521.81
PWY-7229	superpathway of adenosine nucleotides de novo biosynthesis I	21697.02	30215.63	33022.91	24514.54	36879.97	27856.04

Table S5. Average dry weight (g) of *Vigna unguiculata* plants inoculated with different soil samples (mean ± SD). S: Brownish-gray sandy; R: Red soils of medium texture; O: Red soils of medium texture oxic; SL: *Brachystegia* rhizosphere on S with low fire frequency; SH: *Brachystegia* rhizosphere on S with high fire frequency; RL: *Brachystegia* rhizosphere on R with low fire frequency; RH: *Brachystegia* rhizosphere on R with high fire frequency; OL: *Brachystegia* rhizosphere on O with low fire frequency; OH: *Brachystegia* rhizosphere on O with high fire frequency. Uninoculated plants were also included supplied either with nitrogen (TN) or without mineral N (T0). Values of shoot dry weight are the average of replicates/soil type. Es (%) was calculated as Es= (Xs − XT0/XTN − XT0) × 100, where Xs represents the mean dry weight of inoculated shoots ± SD; XTN the mean dry weight of plants with nitrogen control; XT0 the mean dry weight of uninoculated plants.

Soil Type	Fire Frequency	Code	Replicate 1	Replicate 2	Replicate 3	Replicate 4	Replicate 5	Average	Symbiotic %
Sandy soils	Low	SL	0.21	0.31	0.13	0.37	0.23	0.25	189.84
	High	SH	0.09	0.06	0.14	0.17	0.16	0.124	42.18
Red soils	Low	RL	0.41	0.16	0.09	0.09	0.21	0.192	121.87
	High	RH	0.20	0.31	0.13	0.37	0.23	0.248	187.50
Oxi-soils	Low	OL	0.42	0.33	0.05	0.36		0.290	236.71
	High	OH	0.33	0.26	0.08	0.10	0.31	0.216	150.00

Table S6. Taxonomical identification of isolates from *Vigna unguiculata* root nodules inoculated with NSR soil samples and related phylum according to the most related source organism deposited in GenBank. Population codes follow Figure 1. GenBank accession numbers indicate the sequences generated in this study.

Soil type	Fire frequency	Isolate	Most related 16S rRNA gene sequence(s)	Phylum	GenBank accession number	% Identity
Sandy soils	Low	SL3	*Rhizobium* sp. strain LrSB2	Proteobacteria	MZ575037	98.09%
		SL4	*Cohnella* sp. KS7	Firmicutes	MZ575038	88.77%
		SL6	Uncultured bacterium clone PL11D04		MZ575039	92.44%
		SL9B	*Mesorhizobium* sp. OA245	Proteobacteria	MZ575040	96.27%
		SL9C	*Agrobacterium* sp. strain AgSP1	Proteobacteria	MZ575041	93.75%
		SL10AB	*Stenotrophomonas* sp. YAZ112	Proteobacteria	MZ575042	95.59%
		SL10AC	*Stenotrophomonas maltophilia* strain NRB225	Proteobacteria	MZ575043	85.60%
		SL11A	Bacterium strain BS1066		MZ575044	85.24%
	High	SH1	*Xanthomonas oryzae pv. oryzae* strain MTU	Proteobacteria	MZ575045	97.14%
		SH3A	*Herbaspirillum huttiense* Sal 36	Proteobacteria	MZ575046	96.39%
		SH5A	*Pseudomonas* sp. strain S-1	Proteobacteria	MZ575047	92.63%
		SH8	*Xanthomonas oryzae pv. oryzae* strain KRG	Proteobacteria	MZ575048	97.14%
Red soils	Low	RL4A	*Agrobacterium fabrum* strain C58	Proteobacteria	MZ575008	99.80%
		RL5A	*Variovorax guangxiensis* strain GXGD002	Proteobacteria	MZ575009	93.39%
		RL5B	*Agrobacterium salinitolerans* strain YIC 5082	Proteobacteria	MZ575010	91.13%
		RL6A	*Stenotrophomonas maltophilia* strain IAM 12423	Proteobacteria	MZ575011	98.92%
		RL7A	*Rubrimonas shengliensis* strain SL014B-28A2	Proteobacteria	MZ575012	81.63%
		RL8DB	*Lysobacter soli* strain DCY21	Proteobacteria	MZ575013	99.60%
		RL9C	Uncultured bacterium clone Luq_GN460_030		MZ575014	89.59%

		RL10A	Uncultured bacterium clone PL11D04		MZ575015	88.89%
		RL10B	*Rhizobium* sp. NA11040	Proteobacteria	MZ575016	94.17%
		RL10C	*Rhizobium viscosum* strain APP111	Proteobacteria	MZ575017	94.06%
		RL11A	*Rhizobium mesosinicum* strain CCBAU 25010	Proteobacteria	MZ575018	96.32%
		RL11B	*Pseudomonas monteilii* strain NBRC 103158	Proteobacteria	MZ575019	98.87%
		RL13B	*Variovorax* sp. strain UFLA_290	Proteobacteria	MZ575020	90.48%
		RL14A	*Variovorax* sp. strain Beta-76	Proteobacteria	MZ575021	91.02%
		RL14B	*Agrobacterium salinitolerans* strain YIC 5082	Proteobacteria	MZ575022	92.16%
		RL16A	*Neorhizobium galegae* strain NBRC 14965	Proteobacteria	MZ575023	91.21%
		RL17	*Ensifer adhaerens* strain NBRC 100388	Proteobacteria	MZ575024	83.69%
		RL19B	*Variovorax defluvii* strain 2C1-b	Proteobacteria	MZ575025	77.03%
		RL20	*Agrobacterium rhizogenes* strain IFO 13257	Proteobacteria	MZ575026	77.85%
	High	RH5A	*Rhizobium kunmingense* strain LXD30	Proteobacteria	MZ575027	87.10%
		RH5B	*Lactobacillus reuteri* strain NBRC 15892	Firmicutes	MZ575028	89.03%
		RH6A	*Agrobacterium fabrum* strain C58	Proteobacteria	MZ575029	87.23%
		RH8A	*Lactobacillus reuteri* strain K100	Firmicutes	MZ575030	89.66%
		RH9A	*Mesorhizobium* sp. OA245	Proteobacteria	MZ575031	96.27%
		RH11A	*Rhizobium pusense* strain NRCPB10	Proteobacteria	MZ575032	99.02%
		RH12A	*Agrobacterium salinitolerans* strain YIC 5082	Proteobacteria	MZ575033	79.91%
		RH13A	*Mesorhizobium* sp. OA245	Proteobacteria	MZ575034	93.22%
		RH14A	*Ensifer adhaerens* strain NBRC 100388	Proteobacteria	MZ575035	90.34%
		RH18A	*Ensifer adhaerens* strain P55	Proteobacteria	MZ575036	84.37%
Oxi – soils	Low	OL1A	*Pseudomonas* sp. P108	Proteobacteria	MZ574985	97.37%
		OL1B	Bacterium strain TS-3-27		MZ574986	79.92%

	OL2A	*Rhizobium sp*. strain YE2-4	Proteobacteria	MZ574987	98.15%
	OL2B	*Stenotrophomonas maltophilia* strain TCCC11216	Proteobacteria	MZ574988	97.60%
	OL2C	*Stenotrophomonas maltophilia* strain SCS1.1	Proteobacteria	MZ574989	96.21%
	OL3C	Uncultured bacterium clone JSP_109 16S		MZ574990	96.30%
	OL4	*Pseudomonas nitritireducens* strain NRB021	Proteobacteria	MZ574991	87.80%
	OL5A	*Agrobacterium tumefaciens* strain UHFBA-215	Proteobacteria	MZ574992	90.85%
	OL6A	*Rhizobium* sp. strain BR13542	Proteobacteria	MZ574993	95.58%
	OL7A	*Stenotrophomonas* sp. strain EMP41	Proteobacteria	MZ574994	97.55%
	OL10A	*Stenotrophomonas maltophilia* strain BGS15	Proteobacteria	MZ574995	80.88%
	OL12A	*Stenotrophomonas maltophilia*	Proteobacteria	MZ574996	98.36%
	OL13A	Uncultured Stenotrophomonas sp.		MZ574997	94.57%
	OL14A	*Stenotrophomonas maltophilia* strain Sea-8	Proteobacteria	MZ574998	97.33%
	OL15A	*Stenotrophomonas maltophilia* strain ER39	Proteobacteria	MZ574999	97.99%
	OL16	*Stenotrophomonas* sp. strain JDMARP21	Proteobacteria	MZ575000	98.80%
High	OH3BB	Uncultured bacterium clone JSP_70		MZ575001	92.86%
	OH3BC	*Rhizobium* sp. KT24	Proteobacteria	MZ575002	98.53%
	OH7A	*Stenotrophomonas maltophilia*	Proteobacteria	MZ575003	98.46%
	OH9A	Bacterium strain BS1778		MZ575004	95.57%
	OH10A	*Agrobacterium* sp. strain A19T2-60D-R-025 R	Proteobacteria	MZ575005	97.86%
	OH11A	*Mesorhizobium* sp. OA245	Proteobacteria	MZ575006	96.27%
	OH12A	*Agrobacterium* sp. strain NAE168	Proteobacteria	MZ575007	94.95%
